# Factors associated with spoken language comprehension in children with cerebral palsy: a systematic review

**DOI:** 10.1111/dmcn.14651

**Published:** 2020-08-27

**Authors:** Emma Vaillant, Johanna J M Geytenbeek, Elise P Jansma, Kim J Oostrom, R Jeroen Vermeulen, Annemieke I Buizer

**Affiliations:** ^1^ Department of Rehabilitation Medicine Amsterdam Movement Sciences Amsterdam UMC Vrije Universiteit Amsterdam Amsterdam the Netherlands; ^2^ Department of Epidemiology and Biostatistics EMGO+ Institute for Health and Care Research and Medical Library Amsterdam UMC Vrije Universiteit Amsterdam Amsterdam the Netherlands; ^3^ Psychosocial Department Amsterdam Reproduction and Development Emma Children’s Hospital Amsterdam UMC University of Amsterdam and Vrije Universiteit Amsterdam Amsterdam the Netherlands; ^4^ Department of Neurology Maastricht UMC+ Maastricht the Netherlands

## Abstract

**Aim:**

To identify factors that are relevant for spoken language comprehension in children with cerebral palsy (CP), following the International Classification of Functioning, Disability and Health – Children and Youth (ICF‐CY) framework.

**Method:**

A systematic literature search was conducted using the electronic literature databases PubMed, Embase, PsycInfo, and Cochrane Library, from January 1967 to December 2019. Included studies involved children with CP, results regarding spoken language comprehension, and analysis of at least one associated factor. Factors were classified within ICF‐CY domains.

**Results:**

Twenty‐one studies met inclusion criteria. Factors in the ICF‐CY domains of body functions and structure were most frequently reported. White brain matter abnormalities, motor type, functional mobility, and intellectual functioning appear to be relevant factors in spoken language comprehension in CP. Factors in the domain of activities and participation, as well as contextual factors, have rarely been studied in the context of spoken language comprehension in CP.

**Interpretation:**

Most factors known to be important for spoken language comprehension in typically developing children and/or known to be susceptible to change by interventions are understudied in CP.

**Abbreviations**
AAC: Augmentative and alternative communication
CCDI: Chinese Children Developmental Inventory
ICF‐CY: International Classification of Functioning, Disability and Health – Children and Youth

**What this paper adds**

- Factors related to spoken language comprehension are understudied in cerebral palsy.
- Factors in the ICF‐CY ‘body functions and structures’ domain are investigated most.
- Structural brain abnormalities, motor type, functional mobility, and intellectual functions are relevant

In cerebral palsy (CP), motor problems are often accompanied by problems in communication. A strong relation exists between the severity of motor involvement in CP, expressed as Gross Motor Function Classification System (GMFCS) level, and communication problems.^1^, ^2^, ^3^, ^4^ While communication problems are observed in 58% to 81% of children with mild to moderate functional mobility limitations (i.e. GMFCS levels I–III), this rate can rise to 100% in children with most severely affected functional mobility (GMFCS levels IV–V).^4^ Children with communication problems have less fortunate prospects of participation and engagement across a range of activities, including self‐development, learning, and social functioning.^3^, ^5^, ^6^, ^7^

Communication is an overall concept for human interaction. To delineate the scope and impact of communication problems on the functioning of a child with CP, the structure of the International Classification of Functioning, Disability and Health – Children and Youth (ICF‐CY) can be used. The ICF‐CY is a theoretical framework that has been developed by the World Health Organization, and is derived from the ICF.^8^ It conceptualizes functioning and disability as a dynamic interaction between children's health condition, their development, and contextual factors. Contextual factors comprise two components: environmental and personal factors (Fig. 1).

**Figure 1:**
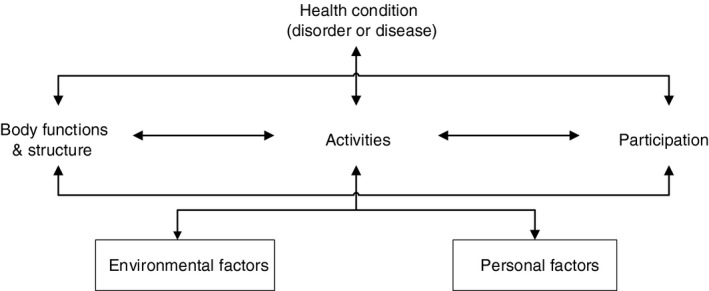
The International Classification of Functioning, Disability and Health – Children and Youth conceptualizes a child’s level of functioning as a dynamic interaction between their health conditions, environmental factors, and personal factors.

At the ‘body function and structure’ level, communication depends on the elements of global intellectual functioning, specific cognitive functions of language, voice, and speech, movement related functions that underlie problems in writing and/or gesturing, and on sensory functions. In the ‘activities and participation’ domain of the ICF‐CY, communication is differentiated into receiving and producing language, conversation, and use of communication device and techniques. Factors that are recognized in the contextual domain are educational level of parents, socioeconomic status, presence of siblings, birth order, language input/caregiver speech, and language activities.^9^, ^10^, ^11^, ^12^, ^13^

Language can be described as a complex system, which makes use of particular rules (syntax). A limited set of sounds or signs can be combined to create an unlimited set of meanings when these rules are used.^14^ The ICF‐CY describes specific cognitive functions of language: recognizing and using signs, symbols, and other components of a language. Spoken language comprehension (receiving language) can be described in the ‘body functions’ domain as grasping the meaning, nature, or significance of a system of conventional spoken language,^15^ and in the ‘activities and participation’ domain, when using spoken language comprehension in daily communication. During typical development, children start to build their spoken language comprehension skills on conceptual representations of objects, relationships, and (daily) activities they are involved in.^16^

In CP, a discrepancy can occur between a child's skills in comprehension and in production of spoken language.^17^, ^18^ Early insight into such a discrepancy is important to support the participation and development of the child. So far, especially production of spoken language has been subject to study in CP. Associations between production of spoken language and different motor types of CP, and between production of spoken language and speech functions have been reported.^19^, ^20^, ^21^, ^22^, ^23^, ^24^, ^25^, ^26^, ^27^, ^28^, ^29^, ^30^ However, studies on the comprehension of spoken language are scarce. It is important to gain knowledge on how contextual factors facilitate or hamper language functioning in children with CP, and how these subsequently impact on communication.

This review aims to identify factors that influence spoken language comprehension in children with CP who experience communication problems, in all ICF‐CY domains. A better understanding of these factors may guide the development of intervention programmes to enhance communication in children with CP.

## Method

### Search strategy

A literature search was performed based on the Preferred Reporting Items for Systematic Reviews and Meta‐Analysis (PRISMA) statement.^31^ To identify all relevant publications, we conducted systematic searches in the bibliographic databases PubMed, Embase, PsycINFO (EBSCO), and the Cochrane Library (Wiley) from inception to 2nd December 2019. Search terms included controlled terms from MeSH in PubMed and Emtree in Embase, as well as free text terms. Free text terms were used only in the Cochrane Library. Search terms expressing ‘cerebral palsy’ (population; children with CP) were used in combination with search terms comprising ‘language development’ (outcome; spoken language comprehension). The references of the identified articles were searched for relevant publications. Duplicate articles were excluded. Only articles written in English were accepted. The full search strategies for all databases can be found in Appendix S1 (online supporting information). The systematic review was registered in PROSPERO (https://www.crd.york.ac.uk/PROSPERO/) in 2017 (registration number: CRD42017075623). In 2019 the date of the literature search was updated from 28th September 2017 (initial search) to 2nd December 2019 (final search). In 2020 contact information of the authors was updated.

### Operational definition

Following Geytenbeek et al.,^32^ we use the term ‘spoken language comprehension (development)’ in this review. Spoken language comprehension is defined as: cognitive and communication functions of decoding spoken messages to obtain their meaning^8^ using a system of conventional spoken language.^15^ In this review, ‘spoken language comprehension’ will include the understanding of a spoken single word (receptive vocabulary), a spoken sentence (syntax comprehension), and understanding spoken discourse (discourse comprehension) in the body functions domain (ICF‐CY: b16700),^33^ and activities and participation domain (ICF‐CY: d3100, d3101).^34^

### Selection process

Two reviewers (EV and JG) independently screened all potentially relevant titles and abstracts for eligibility, using the web application Rayyan QCRI.^35^ If necessary, the full text article was checked for eligibility criteria. Differences in judgement were resolved by discussion between the reviewers. Both reviewers independently read all identified articles selected by described features. Disagreements and/or uncertainty regarding selected articles were resolved by discussion between a larger party of reviewers (EV, JG, AB, KO, JV).

Studies were included if they met the following criteria: (1) published between January 1967 and December 2019; (2) written in English; (3) published in a peer reviewed journal; (4) investigated the association between spoken language comprehension (including alternate terminology of spoken language comprehension) and at least one factor related to the domains of body function and body structure, activities and participation, and/or contextual domain, that is, environmental and/or personal factors (comparison); (5) described which standardized language test or assessment/questionnaire/scale was used (intervention); (6) reported results and data relating to spoken language comprehension; and (7) at least one of the participants in the study sample was a child with CP aged 0 to 18 years; individual or group results of children with CP were specifically reported.

Studies were excluded when: (1) they were written in a language other than English; (2) the focus was to investigate expressive language, and/or communication skills in general, literacy skills, expressive vocabulary, expressive morphology, expressive syntax, and pragmatic skills; (3) non‐standardized language assessments/tests/scales/questionnaires were used; (4) relationship(s) between spoken language comprehension and a factor within the ICF‐CY domains were not investigated and/or reported; (5) only general information about CP and language (comprehension) development was described; and (6) it was one of the following publication types: letter, commentary, presentation, or editorial.

A data extraction form was created to ensure adequate and reliable data extraction of all identified studies from the electronic search. In line with the ICF‐CY domains^8^ and the ICF‐CY core sets for children with CP described by Schiariti et al.,^36^ the study characteristics for data extraction were: aim of the study, design, number of participants, age range, sex, structure of brain (ICF‐CY code: s110), motor type and distribution (s110), spoken language comprehension outcome and used test and/or scale and/or questionnaire (b16700 or d3100,d3101), and outcome measures in relation with at least one of the potential associated factors within the ICF‐CY domains (i.e. ‘body function and body structure’, ‘activities and participation’, and ‘contextual domain’, consisting of environmental and personal factors; Table 1). The method of measurement of the potential associated factors was also documented. These potential associated factors are related to CP specifically, or are known to be relevant for spoken language comprehension in typically developing children.

**Table 1:** ICF‐CY domains of potential associated factors with spoken language comprehension

**Body structure** **Structure of brain** (s110) Motor type (s110) Motor distribution (s110) Epilepsy (s110)	**Body function** **Intellectual functions** (b117) **Specific cognitive functions of expressive language** (b1671) **Seeing functions** (b210) **Hearing functions** (b230) **Speech functions** **(yes or no)** (b320)		**Activities and participation** **Reading skills** (d166) **Writing and spelling skills** (d170) Speech production (VSS) (d330) **Communication** (including CFCS) (d350) Arm and hand functioning (including MACS) (d440,445) Mobility (including GMFCS) (d450,455,460,465) **Social skills** (d710,720,750) **Language activities** (d810) **Preschool education** (d815) **School education** (d820) **Symbolic play** (d880)
**Contextual domain**			
**Environmental factors** AAC system (e125) **SES (e165)** **Educational level of the parents** (e165) **Number of siblings and birth order** (e310) **Language input/caregiver speech** (e410) **Preschool education and school education** (e585)		**Personal factors** **Age** **Sex**	

Factors that are generally considered relevant to spoken language comprehension in typically developing children are shown in bold type. ICF‐CY, International Classification of Functioning, Disability and Health – Children and Youth; VSS, Viking Speech Scale; CFCS, Communication Function Classification System; MACS, Manual Ability Classification System; GMFCS, Gross Motor Function Classification System; AAC, augmentative and alternative communication; SES, socioeconomic status.

### Quality assessment and risk of bias

The full text of the selected articles was obtained for further review. Both reviewers (EV and JG) independently evaluated the methodological quality of the full text papers to determine quality including risk of bias of the individual studies. Because most rating criteria for quality appraisal are limited to randomized or non‐randomized studies, or are not appropriate for use because of difficulties in determining equivalence between different methodologies, a modified version of Downs and Black's quality assessment form,^37^ with an addition of level of evidence classification based on Perera,^38^ was used (Appendix S2, online supporting information). The performance of the Downs and Black quality assessment was shown to be reliable with a high internal consistency, a good test–retest and inter–intra rater reliability, and a high criterion validity.^37^

Articles were assessed on 13 quality items listed in Appendix S2, with 1 indicating that the quality was adequate and 0 indicating not adequate or not possible to determine. The design of the studies was also assessed on classification of level of evidence: a maximum score of 4 was obtained for strong study designs and a minimum score of 1 for weak study designs. Total scores were calculated as score of classification of level of evidence plus item scores. The potential total score was calculated as maximum score of classification of level of evidence (maximum of 4) plus highest obtainable item score (maximum of 13) minus total of non‐applicable scores. Percentage was calculated as total score divided by potential total score, multiplied by 100%. Higher scores indicate a better methodological quality of the study and a lower risk of bias. Cut‐off percentages, as suggested by Hooper et al.,^39^ were used to categorize studies by quality: excellent (91–100%); good (71–90%); fair (51–70%); and poor (≤50%). Funding sources and potential conflict of interests of individual studies were documented.

## Results

### Search results

The literature search generated a total of 4224 references. After removing duplicates of references that were selected from more than one database, 2898 references remained. After the selection based on title and abstracts, 72 studies remained for full text reading and reviewing references. Twenty‐one studies finally met the inclusion criteria (see Fig. S1, online supporting information, for the flow chart of the search and selection process).

Excluded studies did not specify spoken language comprehension outcomes (*n*=20), did not test spoken language comprehension at all or used a non‐standardized test (*n*=12), or did not describe an association with any factor(s) within the domains of the ICF‐CY (*n*=7). Other excluded studies did not differentiate the language outcomes between children with CP and/or children with another medical diagnosis or typically developing children (*n*=5), reported about overall communication in or overall information about children with CP (*n*=6), or full text was not retrievable (*n*=1).

### Study and participant characteristics

The 21 included studies involved 1815 participants diagnosed with CP; the age range varied from 16 months to 24 years. Most participants had spastic CP, and half of the participants had bilateral CP. The majority of the included studies (*n*=14) used the GMFCS to describe functional mobility, in five studies functional mobility was otherwise specified, and in two studies it was not reported. Study populations ranged from a group of eight participants to a cohort of 418 participants. See Table 2 for participant characteristics of the included children.

**Table 2:** Participant characteristics of included children

Age range	16mo–24y
Total	1815 (100%)
*Motor type*	
Spastic	1379 (75.9%)
Non‐spastic	
Dyskinetic	159 (8.8%)
Ataxic	14 (0.8%)
Not specified	88 (4.8%)
Mixed CP	25 (1.4%)
Unknown/not reported	22 (1.2%)/128 (7.1%)
*Motor distribution*	
Unilateral	492 (33.9%)
Bilateral	925 (51.0%)
Not reported	398 (21.9%)
*GMFCS level*	
I	489 (33.9%)
II	190 (13.2%)
III	175 (12.1%)
IV	269 (18.7%)
V	319 (22.1%)
Not specified/not reported	314 (17.3%)/59 (3.2%)

### Quality assessment and risk of bias

The quality rates of the 21 included studies ranged between 33% and 94%. Two studies had an ‘excellent’ quality level,^1^, ^40^ 14 studies scored ‘good’,^17^, ^18^, ^41^, ^42^, ^43^, ^44^, ^45^, ^46^, ^47^, ^48^, ^49^, ^50^, ^51^, ^52^ three studies ‘fair’,^53^, ^54^, ^55^ and two studies ‘poor’.^56^, ^57^ With the exception of one,^57^ all studies scored ‘good’ on the items referring to reporting. Four studies^53^, ^54^, ^56^, ^57^ did not report actual probability values for the main outcomes. The items about external validity showed that in two studies^54^, ^57^ the individuals who agreed to participate were not representative of the entire population from which they were recruited and in 12 studies^17^, ^18^, ^41^, ^42^, ^44^, ^45^, ^46^, ^47^, ^48^, ^53^, ^56^, ^57^ it was not possible to determine whether this was the case. The items concerning internal validity (bias) showed that in all but one study^57^ it was made clear if the results of the study were based on data dredging. Overall, statistical tests used to assess the main outcomes were appropriate and main outcome measures used were valid and reliable, except for one study.^56^ None of the studies reported a power analysis. Five studies^41^, ^42^, ^48^, ^52^, ^57^ did not describe their funding sources and 10 studies^1^, ^41^, ^42^, ^45^, ^48^, ^52^, ^54^, ^55^, ^56^, ^57^ did not state whether there were potential conflicts of interest. The results of the quality assessment are shown in Appendix S3 (online supporting information).

### Factors associated with spoken language comprehension

See Table S1 (online supporting information) for an overview of the 21 studies and reported factors, according to ICF‐CY domains.

#### Body structure and body function domain

##### Structural brain abnormalities (ICF‐CY: s110)

Three studies investigated brain abnormalities using magnetic resonance imaging (MRI) characteristics in association with spoken language comprehension.^42^, ^43^, ^44^ One study used a mixed sample of toddlers with CP (*n*=131),^43^ one study used a sample of preschool children (aged 2–5y) with bilateral spastic CP (*n*=46),^42^ and one study described spoken language comprehension in a wider sample of children with spastic (*n*=43) and dyskinetic (*n*=37) CP aged 1 year 7 months to 12 years (GMFCS levels IV and V).^44^ All these studies found a significant association between spoken language comprehension and severity of periventricular leukomalacia.^42^, ^43^, ^44^ More specifically, global left as well as right hemisphere white matter lesions or lesions to the presumed language pathway were reported as factors significantly related to spoken language comprehension.^43^

In a sample of non‐speaking children with severe CP (GMFCS levels IV and V), thinning of the corpus callosum in children with basal ganglia necrosis and parieto‐occipital white matter reduction in children with periventricular leukomalacia were related to poor spoken language comprehension. In children with miscellaneous patterns of brain abnormalities, associations between MRI pattern and spoken language comprehension were inexplicit.^44^ In one study, no association was found between the MRI pattern of brain malformations or basal ganglia necrosis and spoken language comprehension in a mixed sample of toddlers with CP at all GMFCS levels.^43^

##### Motor type (ICF‐CY: s110)

Six studies investigated motor type in association with spoken language comprehension.^1^, ^17^, ^18^, ^40^, ^42^, ^52^ Studies in a mixed sample of toddlers and schoolchildren with severe CP (GMFCS levels IV and V) showed that children with dyskinetic CP performed significantly better than children with spastic CP.^17^, ^18^ Spoken language comprehension was age‐appropriate in 50% of the children with dyskinetic CP, as opposed to 8% of children with spastic CP.^18^ Three other studies could not establish significant associations between motor type of CP and spoken language comprehension.^1^, ^42^, ^52^ These were studies in mixed samples of toddlers and schoolchildren with CP at all GMFCS levels (*n*=131,^1^ *n*=172,^42^ *n*=70^52^). One study studying a mixed sample of schoolchildren and adolescents with CP (*n*=418) at all GMFCS levels could not find any differences in the developmental trajectories of spoken language comprehension between groups of patients with different motor types of CP.^40^

##### Motor distribution (ICF‐CY: s110)

Five studies investigated motor distribution in association with spoken language comprehension.^1^, ^42^, ^48^, ^52^, ^57^ In a study with a mixed sample of children with CP between 1 year 6 months and 5 years 8 months (*n*=137, mobility expressed in Chinese Children Developmental Inventory [CCDI], subsection Gross Motor), children with spastic quadriplegia had poorer spoken language comprehension than children with spastic diplegia.^48^ When differentiating between unilateral versus bilateral CP in children between 3 and 7 years, a significant association was shown with spoken language comprehension in univariate analysis in which children with unilateral CP performed better than children with bilateral CP, at all GMFCS levels (*n*=172).^42^ However, this was no longer the case in multivariate analysis where cognitive functioning appeared to be more important.^42^ Furthermore, no difference in spoken language comprehension results was found between left and right unilateral CP, in a sample with children with unilateral CP, motor type not further specified, between 2 and 18 years (*n*=80).^57^ One study in a sample of toddlers and schoolchildren with CP at all GMFCS levels (*n*=131) reported a significant association between motor distribution (unilateral and bilateral CP) and a significant association between three and four limbs involved (compared to two limbs involved) with spoken language comprehension in univariate analysis.^1^ However, these associations were not found in multivariate analyses for spoken language comprehension where GMFCS level and preterm birth status was found to be more important. Another study did not find an association between motor distribution and spoken language comprehension in a mixed sample of children with CP at all GMFCS levels (*n*=70).^52^

##### Epilepsy (ICF‐CY: s110)

Results of four studies suggested no association between epilepsy and spoken language comprehension.^1^, ^17^, ^18^, ^42^ These studies were performed in different samples: a mixed sample of toddlers with CP (*n*=124) at all GMFCS levels^1^ and mixed samples of toddlers and schoolchildren (*n*=68,^17^ *n*=87,^18^ both at GMFCS levels IV and V; and *n*=172,^42^ at all GMFCS levels). Three studies^1^, ^17^, ^18^ only mentioned the presence or absence of epilepsy but did not test associations. One study shared more detailed information on epilepsy, that is whether a child had no seizures (*n*=103), controlled epilepsy without antiseizure medication (*n*=22), controlled epilepsy with antiseizure medication (*n*=30), or epilepsy with antiseizure medication or epileptic surgery (*n*=17).^42^ However, this last study did not test whether epilepsy was an explanatory factor for spoken language comprehension in their mixed sample of children with CP between 3 and 7 years (*n*=172) at all GMFCS levels.^42^

##### Intellectual functioning (ICF‐CY: s117)

Six studies^40^, ^42^, ^50^, ^55^, ^56^, ^57^ reported a significant association between intellectual functioning and spoken language comprehension. Lower intelligence level coincided with poorer language comprehension,^40^, ^42^, ^50^, ^55^, ^56^, ^57^ in mixed samples of children between 3 and 7 years (*n*=172) at all GMFCS levels,^42^ between 5 and 6 years (*n*=84) at all GMFCS levels,^50^ between 1 year 10 months and 9 years (*n*=36, using the Gross Motor Limitation Scale, all levels),^55^ a sample of children between 2 and 18 years with unilateral CP (*n*=80),^57^ a mixed sample of children and adolescents between 0 and 24 years (*n*=418) at all GMFCS levels,^40^ and a sample of teenagers with CP (*n*=48, motor type and mobility not further specified).^56^ One study described an interaction between motor type, intellectual functioning, and spoken language comprehension:^40^ in children with unilateral spastic CP, no differences in language comprehension trajectory levels were found between children with and without intellectual disability, but in children with bilateral spastic CP or non‐spastic CP, lower and less favourable language comprehension trajectories were found for children with intellectual disability.^40^

##### Specific cognitive functions of expressive language (ICF‐CY: b1671)

Three studies^41^, ^48^, ^51^ reported expressive language to be an important prerequisite for language comprehension. Two studies on toddlers with bilateral spastic CP (*n*=46,^41^ *n*=137^48^) found a significant interaction on the CCDI subsection of Expressive Language and Concept Comprehension, amongst other significant correlations between developmental function measures. In a small mixed sample of school children with CP (*n*=15, GMFCS levels I–IV), one study found a significant association between narrative abilities and receptive grammar.^51^

##### Sensory functions (seeing functions [ICF‐CY: b210] and hearing functions [ICF‐CY: b230])

One study reported absence of an association between hearing and spoken language comprehension in a mixed sample of toddlers with CP (*n*=124) at all GMFCS levels.^1^ Investigated in two studies,^1^, ^42^ no association was found between visual functions and spoken language comprehension in a mixed sample of toddlers with CP (*n*=124) at all GMFCS levels^1^ and a mixed sample of children with CP between 3 and 7 years (*n*=172) at all GMFCS levels.^42^ In one study there was a significant association between vision and spoken language comprehension in univariate analysis, but this association did not hold in multivariate analysis where it was overruled by another factor (i.e. functional mobility).^1^

##### Speech functions (ICF‐CY: b320)

Five studies reported a significant association between speech functions and spoken language comprehension in mixed samples of toddlers and children with CP with different motor speech skills.^46^, ^47^, ^49^, ^53^, ^56^ Speaking children had better spoken language comprehension than non‐speaking children, in a small mixed sample of toddlers with CP (*n*=8, GMFCS levels I, III, and V)^53^ and a sample of toddlers with spastic CP (*n*=30) at all GMFCS levels.^47^ Motor speech problems were associated with impairment in spoken language comprehension, in a mixed sample of toddlers with CP (*n*=71) at all GMFCS levels,^49^ a sample of toddlers with spastic CP (*n*=30) at all GMFCS levels,^47^ and a sample of teenagers with CP (*n*=48, motor type and mobility not further specified).^56^ In the latter study motor speech problems were not associated with grammatical competence (i.e. comprehension of the sentence syntax) in teenagers with CP, with or without understandable speech.^56^

#### Activity and participation domain

##### Reading skills (ICF‐CY: d166)

Two studies investigated the association between reading skills and spoken language comprehension.^45^, ^54^ Spoken language comprehension seemed to be an important ability for inferential reading (i.e. the ability to understand the underlying meaning of a text).^45^ In one study, a significant association was found between inferential comprehension and sentence comprehension, and between literal comprehension and receptive vocabulary, in a sample of schoolchildren with spastic diplegia (*n*=10, mobility not reported).^45^ However, another study found that reading skills were not associated with receptive vocabulary in a mixed sample of schoolchildren with CP (*n*=15: wheelchair users [*n*=2] and in a group where functional mobility was not described [*n*=13]).^54^

##### Arm and hand functioning (ICF‐CY: d440, 445)

Five studies investigated arm and hand functioning in association with spoken language comprehension.^17^, ^18^, ^41^, ^48^, ^57^ Two studies used the Manual Ability Classification System^17^, ^18^ and two studies used the Fine Motor ability subsection of the CCDI^41^, ^48^ to define arm and hand functioning. One study defined hand functioning based on grip and use of the hand.^57^ In two studies, no significant associations were reported between arm and hand functioning reported as Manual Ability Classification System level and spoken language comprehension in a mixed sample of children between 1 years 7 months and 12 years (*n*=68)^17^ and between 1 years 9 months and 12 years (*n*=87;^18^ GMFCS levels IV and V).^17^, ^18^ In the study which used their own classification based on grip and use of the hand, no association was found between hand functioning and spoken language comprehension in a sample of children with unilateral CP between 2 and 18 years (*n*=80).^57^ However, fine motor skills classified with the CCDI showed a significant association with spoken language comprehension in children between 2 and 5 years with bilateral spastic CP (*n*=46, mobility expressed in CCDI, subsection Gross Motor),^41^ and a mixed sample of children between 1 years 6 months and 5 years 8 months (*n*=137, mobility expressed in CCDI, subsection Gross Motor).^48^

##### Functional mobility (ICF‐CY: d450, 455, 460, 465)

Twelve studies reported a significant association between functional mobility and spoken language comprehension in mixed samples of children with CP at all GMFCS levels.^1^, ^17^, ^18^, ^41^, ^42^, ^43^, ^48^, ^49^, ^50^, ^52^, ^55^, ^57^ However, in three studies^42^, ^50^, ^57^ an association between mobility and spoken language comprehension could not be demonstrated in mixed samples of toddlers and school children (*n*=172,^42^ *n*=84^50^) both at all GMFCS levels, and in a sample of children between 2 and 18 years with unilateral CP (*n*=80).^57^ Gross motor skills classified with CCDI subsection Gross Motor in a sample of toddlers with bilateral spastic CP (*n*=46,^41^ *n*=137^48^) and with the Gross Motor Limitation Scale (all levels) in a sample of children between 1 years 10 months and 9 years with spastic CP also showed a significant association with spoken language comprehension.^55^

##### Social skills (ICF‐CY: d710, 720, 750)

Three studies reported positive significant associations between social skills and spoken language comprehension.^41^, ^48^, ^49^ One study focused on a mixed sample of children with CP (*n*=71) at all GMFCS levels and assessed language comprehension, with subsection Symbolic Communication of the Communication and Symbolic Behavior Scales ‐ Developmental Profile infant‐toddler checklist at the age of 24 months, and social functions, with the subsection Social Function of the Pediatric Evaluation of Disability Inventory at the age of 60 months.^49^ Two studies assessed spoken social skills with the CCDI subsection Personal‐Social, and spoken language comprehension with the CCDI subsection Concept Comprehension, in samples of toddlers with CP; one mixed sample (*n*=137)^48^ and one sample with children with bilateral spastic CP (*n*=46).^41^ Better spoken language comprehension contributed to better social skills, and better social skills contributed to better spoken language comprehension.

#### Contextual factors: environmental and personal factors

##### Socioeconomic status (ICF‐CY: e165)

Reported in only one study, no significant association was found between socioeconomic status and spoken language comprehension in a mixed sample of toddlers with CP (*n*=124) at all GMFCS levels.^1^

##### Parental educational level (ICF‐CY: e165)

In one study, no significant association was found between educational level of the parents and spoken language comprehension measured with Computer‐Based instrument for Low motor Language Testing in a sample with children between 1 years 7 months and 12 years with spastic (*n*=31) and dyskinetic CP (*n*=37, GMFCS levels IV and V).^17^

##### Birth order (ICF‐CY: e310)

One study explicitly studied the effect of birth order on language comprehension but could not establish an association in multivariate analysis.^1^

##### Age (ICF‐CY: contextual domain, personal factor)

Three studies investigated the association between age and spoken language comprehension.^17^, ^18^, ^56^ One study, in a sample with children with unspecified CP between 10 and 18 years (*n*=48, mobility not specified), reported no significant association between age and spoken language comprehension.^56^ However, in two studies,^17^, ^18^ a significant positive association was found between chronological age and spoken language comprehension in a mixed sample of children between 1 year 7 months and 12 years (*n*=68)^17^ and between 1 year 9 months and 12 years (*n*=87,^18^ GMFCS levels IV and V in both studies). Chronological age was found to be a positive modulating factor for severe CP and more complex sentence comprehension.^17^

##### Sex (ICF‐CY: contextual domain, personal factor)

Reported in one study, no significant differences were found in sentence comprehension between males and females in a mixed sample of children with CP between 1 year 7 months and 12 years (*n*=68, GMFCS levels IV and V).^17^

### Factors not reported

Not all of the factors associated with spoken language comprehension (development) that we know in typically developing children have been the subject of study in CP. These factors all pertain to the activities and participation domain and the contextual environmental factors.

In the activities and participation domain, the relationship between spoken language comprehension and writing and spelling skills (ICF‐CY: d170), speech (classified with the Viking Speech Scale; ICF‐CY: d330), language activities (ICF‐CY: d810), preschool education (ICF‐CY: d815), school education (ICF‐CY: d820), and symbolic play (ICF‐CY: d880) were not reported in the included studies.

In the contextual domain, more specifically the environmental factors, the effect of an augmentative and alternative communication (AAC) system (ICF‐CY: e125), number of siblings (ICF‐CY: e310), language input/caregiver speech (ICF‐CY: e410), and preschool and school education of the child (ICF‐CY: e585) were not reported in the included studies.

## Discussion

This review aimed to identify factors relevant for spoken language comprehension in children with CP and accompanying communication problems. Following the ICF‐CY framework, factors in the domains of ‘body function and structure’ have been the subject of study and shown to be relevant. Factors in the domains of ‘activities and participation’ and contextual factors have received substantially less attention in the literature.

### Body function and structure domain

In the ‘body function and structure’ domains, in all but one study structural brain abnormalities were found to be associated with spoken language comprehension. In these three studies the quality of the evidence was good.^42^, ^43^, ^44^ In particular, damage to the white brain matter appears to have a detrimental effect, with the more severe the periventricular leukomalacia, the poorer the language comprehension. Associations between structural damage of specific brain structures, and language problems are suggested for certain subgroups of CP,^42^, ^43^, ^44^ but these findings need replication in larger samples.

The role of motor type of CP has been examined by studies of good^17^, ^18^, ^42^, ^52^ and excellent quality.^1^, ^40^ In severe CP (GMFCS levels IV and V), children with spastic CP have been shown to be at a disadvantage compared to children with dyskinetic CP. For other GMFCS levels this has not been reported. It is not clear whether children with bilateral CP are at a disadvantage compared to children with unilateral CP. Studies on motor distribution are difficult to compare and outcomes are not unanimous, partly because motor distribution is reported and operationalized in various ways; three studies with excellent,^1^ good,^42^ and poor^57^ quality used unilateral and bilateral, two studies with good quality used hemiplegia, diplegia, and quadriplegia.^48^, ^52^ In many studies, motor distribution was not specified for all included children.^17^, ^18^, ^44^, ^47^, ^49^, ^51^, ^52^, ^53^, ^54^, ^56^

The presence of epilepsy was not found to be a factor of decisive influence on spoken language comprehension in children with CP in available good to excellent quality studies.^1^, ^17^, ^18^, ^42^ This is in contrast with research that has shown that significantly more children without CP with active epilepsy and without intellectual disabilities had language scores one standard deviation below average than an age‐matched comparison group without epilepsy.^58^ In children with CP, other factors seem more important for spoken language comprehension, such as underlying brain lesion, severity, or motor type of CP.^18^ However, data exist that supports the hypothesis that language development in CP is jeopardized more seriously in children who suffer epilepsy.^59^ It is possible that epilepsy plays a moderating rather than an immediate role in spoken language comprehension in children with CP. In addition, details on whether the epilepsy was still present with or without the use of antiseizure medication or epileptic surgery was reported in only one study.^42^ Due to adequate medication, it is possible that the influence of epilepsy on language comprehension outcomes is reduced.

Intellectual functioning seems to be strongly related to spoken language comprehension, with better language comprehension in children with a higher level of intelligence,^40^, ^42^, ^50^, ^55^, ^56^, ^57^ with good to excellent quality evidence in three studies.^40^, ^42^, ^50^ However, in one study of excellent quality, this association became less obvious when other parameters such as motor type and distribution of CP were entered into the statistical models.^40^ It is important to realize that determining cognitive abilities accurately in children with CP remains a challenge.^60^

Language production appears to be related positively to language comprehension, in children with CP, with good quality evidence.^41^, ^48^, ^51^ Children with CP and intact speech function have been shown to have better language comprehension than those with accompanying speech function problems.^46^, ^47^, ^49^, ^53^, ^56^ However, speech functions are described differently, limiting the comparison of outcomes between these different studies and thus the role of speech functions and language comprehension. Moreover, studies also showed that age‐appropriate language comprehension skills can exist in the absence of speech.^17^, ^18^, ^56^ Usage of the Viking Speech Scale^61^ may improve description and comparison of speech production in children with CP in future studies.

There is no evidence in available studies that language comprehension in CP^1^, ^42^ is associated differently with sensory functions such as seeing or hearing than in other children.^62^, ^63^ Although the evidence from these studies is of good to excellent quality, sensory function has scarcely been studied in children with CP, and certainly not in relation to language comprehension development. To fully understand the association between sensory function and spoken language comprehension in children with CP, more research is needed.

### Activities and participation domain

In the domain of ‘activities and participation’, the functional mobility level is especially important for spoken language comprehension in CP^1^, ^17^, ^18^, ^41^, ^42^, ^43^, ^48^, ^49^, ^50^, ^52^, ^55^, ^57^ (mostly good quality studies). In addition to considering functional mobility as a proxy of the underlying neurological problems causing CP, children who are less mobile have reduced abilities to explore their environment and to learn by experience.^14^ One possibility could be that certain children are more dependent on what their immediate environment offers them. Consequently, this could influence their overall language development including the development of spoken language comprehension.

A relation between manual function and spoken language comprehension was established as well as rejected in four studies with good quality,^17^, ^18^, ^41^, ^48^ depending on the exact sample under study as well as on the operationalization of the concept of manual function. Accordingly, a definite conclusion on the overall findings remains difficult as yet.

In typical development, communication‐related factors in the domain of activities and participation, such as language activities and symbolic play,^9^, ^10^, ^11^, ^12^, ^13^ are shown to be relevant. This could be of importance for children with CP because most of these factors can be influenced by intervention. Therefore, more thorough knowledge about these factors and how they might impact on language comprehension in children with CP is essential. As in typical development, social skills seem to be relevant in CP, reported in three good quality studies,^41^, ^48^, ^49^ in the sense that social skills are positively related to language comprehension in CP.

Spoken language comprehension could be relevant for reading acquaintance in children with CP; however this statement is based on only two small studies with good^45^ and fair^54^ quality that need replication.

### Contextual domain (personal and environmental factors)

In typical development, age is an important factor for growth in language comprehension skills. The role of age in CP was investigated only in three studies: two with good quality^17^, ^18^ and one with poor quality.^56^ Comparable with typical development, older children with CP have better spoken language comprehension skills.^17^, ^18^, ^56^

No statistically significant associations have been found between spoken language comprehension and contextual factors in CP, such as socioeconomic status (reported in a study of excellent quality),^1^ educational level of the parents (reported in a study of good quality),^17^ birth order,^1^ and sex.^17^ Several environmental factors are considered important for language comprehension in typically developing children but have not yet been the subject of study in CP. For instance, no studies in CP have reported the effect of contextual factors such as number of siblings, language input/caregiver speech, and type of education. Also, the effects of AAC systems on language comprehension have not been studied. Similar to factors in the activities and participation domain, communication‐related factors in the contextual domain (i.e. language input/caregiver speech, AAC system) can be influenced by intervention.

### Limitations of the study

The overall results of the quality ratings showed that the majority of the studies had an excellent or good quality level.^1^, ^17^, ^18^, ^40^, ^41^, ^42^, ^43^, ^44^, ^45^, ^46^, ^47^, ^48^, ^49^, ^50^, ^51^, ^52^ However, five studies had a fair or poor quality level,^53^, ^54^, ^55^, ^56^, ^57^ and therefore in these studies risk of bias is increased. Four of these studies used a cross‐sectional study design^54^, ^55^, ^56^, ^57^ and one study used a retrospective cohort study design^53^ which are both not the most desirable study designs for investigating aetiology.^38^ In aetiology hierarchy, the prospective cohort study is seen as the strongest observational study design^64^ and is therefore the most desirable study design for this purpose.^38^

The results of the quality ratings for external validity suggest that in more than half of the studies it was not possible to determine whether the participants who were prepared to participate were representative of the entire population they were recruited from.^17^, ^18^, ^41^, ^42^, ^44^, ^46^, ^47^, ^48^, ^53^, ^56^, ^57^ Some of the studies included only a small controlled subgroup of the entire population, that is, only children with unilateral CP,^57^ spastic CP,^41^, ^45^, ^48^ or GMFCS levels IV and V.^17^, ^18^, ^44^ Clinical relevance of the outcomes in the studies can be poor for the entire population of children with CP; we should therefore be careful with extrapolate data to other subgroups of the population.

None of the included studies reported a power analysis. It is therefore difficult to determine the statistical and clinical relevance of the outcomes of the individual studies. However, despite the fact that only a minority of the identified studies used a cohort study design^1^, ^41^, ^46^, ^47^, ^53^ which leads to more valid outcomes,^38^ the overall results of the quality assessment yielded studies of good quality. This strengthens our findings, and allows us to draw conclusions of which factors, in the different domains of the ICF‐CY, are relevant for language comprehension development in children with CP and which factors are understudied.

## Conclusion

This systematic review has identified relevant factors, within the different domains of the ICF‐CY, for spoken language comprehension in children with CP, that is, structural brain abnormalities, motor type, motor distribution, intellectual functioning, expressive language, speech, arm and hand functioning, and functional mobility. However, the majority of factors that are known to be important for spoken language comprehension in typically developing children and/or that can be influenced by interventions are understudied in CP. More research on the association between these factors and spoken language comprehension in children with CP is needed for future clinical practice, especially to improve the quality of interventions for children with CP.

## Clinical implications

In speech language pathology practice, most intervention methods for communication that are used have not been developed specifically for children with CP, and lack evidence for this group.^65^ This review shows that little is known about which factors are specifically important to intervene on.^66^ AAC services are commonly considered for children with complex communication needs and many AAC options are available. For further development of effective AAC use, a more profound insight in factors that are relevant, and susceptible to change, in children with CP is warranted.

Reliable testing of spoken language comprehension, even in children with the most severe motor problems, is necessary, and for this, feasible instruments have become available.^18^ More knowledge about communication related factors (such as language activities, language input/caregiver speech, and use of AAC), that can be influenced by speech and language therapy is needed. This will help to improve the design and implementation of interventions, aimed at enhancing spoken language comprehension and overall communication skills in children with CP.

## Future directions for research

More research is necessary to gain insight into factors associated with spoken language comprehension in childhood CP. Valid measures to assess spoken language comprehension in children with CP are few, and have only been recently developed for assessment in children with the most severe motor limitations.^52^, ^67^

Regarding body function and structure, the effect of motor type (i.e. dyskinetic or spastic CP) needs further study. Although only reported in non‐speaking children in GMFCS levels IV and V, motor type is possibly of more importance for spoken language comprehension than GMFCS level.^17^, ^18^ Future research should focus more on the differences between these types of CP and whether these differences are also seen in lower GMFCS levels (i.e. GMFCS levels I–III). Whether epilepsy has an independent effect on spoken language comprehension in CP remains to be determined.

Outcomes in the contextual domain need to be given more attention in future research. Important factors to investigate are symbolic play, language input/caregiver speech, language activities, preschool and school education, expressive language skills, and speech. Also, it would be valuable to gain evidence of how children with CP benefit from AAC; not only in terms of participation and engagement across a full range of environments, but also in terms of spoken language comprehension.

Future research would benefit from longitudinal cohort studies including children in all GMFCS levels and across a broad age range.^66^ To assess spoken language comprehension development and influencing factors, it is recommended to use standardized tests, and to document the potentially associated factors at different measurement points.

## Supporting information


**Appendix S1:** Full search strategies for all databases.

**Appendix S2:** Quality assessment.

**Appendix S3:** Results of the quality assessment.

**Figure S1:** Flowchart of search and selection process.

**Table S1:** Study characteristics and reported factors
