# Supplementary material for: Factors associated with spoken language comprehension in children with cerebral palsy: a systematic review
Source: Dev Med Child Neurol. 2020 Aug 27;62(12):1363–73. doi: 10.1111/dmcn.14651 (PMC7692918; doi:10.1111/dmcn.14651)
Supplement: Supplementary file 1 — Appendix S1: Full search strategies for all databases. [file DMCN-62-1363-s001.docx]

**Appendix S1**

Search strategy in PubMed December 2, 2019 (read from bottom-up).

| **Set** | **Search terms** | **Result** |
| --- | --- | --- |
| #3 | #1 AND #2 | 1384 |
| #2 | "Language Development"[Mesh] OR ((Language[tiab] OR verbal[tiab] OR "Communication"[Mesh] OR communicat*[tiab]) AND (development*[tiab] OR training[tiab] OR learning[tiab] OR function*[tiab] OR participat*[tiab] OR abilit*[tiab])) | 267048 |
| #1 | "Cerebral Palsy"[Mesh] OR cerebral pals*[tiab] OR brain pals*[tiab] OR brain paralys*[tiab] OR central pals*[tiab] OR central paralys*[tiab] OR cerebral paralys*[tiab] OR cerebral pares*[tiab] OR (encephalopathi*[tiab] AND infantil*[tiab]) OR spastic diplegi*[tiab] OR little disease*[tiab] OR little's disease*[tiab] | 33901 |

Search strategy in Embase.com December 2, 2019 (read from bottom-up).

| **Set** | **Search terms** | **Result** |
| --- | --- | --- |
| #4 | #3 AND ('article'/it OR 'article in press'/it OR 'letter'/it OR 'review'/it) | 1753 |
| #3 | #1 AND #2 | 2757 |
| #2 | 'language development'/exp OR ((language:ti,ab OR verbal:ti,ab OR 'interpersonal communication'/exp OR communicat*:ti,ab) AND (development*:ti,ab OR training:ti,ab OR learning:ti,ab OR function*:ti,ab OR participat*:ti,ab OR abilit*:ti,ab)) | 424402 |
| #1 | 'cerebral palsy'/exp OR 'cerebral pals*':ti,ab OR 'brain pals*':ti,ab OR 'brain paralys*':ti,ab OR 'central pals*':ti,ab OR 'central paralys*':ti,ab OR 'cerebral paralys*':ti,ab OR 'cerebral pares*':ti,ab OR (encephalopathi*:ti,ab AND infantil*:ti,ab) OR 'spastic diplegi*':ti,ab OR 'little disease*':ti,ab OR 'littles disease*':ti,ab | 42407 |

Search strategy in PsycINFO December 2, 2019 (read from bottom-up).

| **Set** | **Search terms** | **Result** |
| --- | --- | --- |
| #4 | #3 AND Limiters - Publication Type: All Journals | 821 |
| #3 | #1 AND #2 | 934 |
| #2 | DE "Cerebral Palsy" OR TI ("cerebral pals*" OR "brain pals*" OR "brain paralys*" OR "central pals*" OR "central paralys*" OR "cerebral paralys*" OR "cerebral pares*" OR (encephalopathi* AND infantil*) OR "spastic diplegi*" OR "little disease*" OR "little's disease*") OR AB ("cerebral pals*" OR "brain pals*" OR "brain paralys*" OR "central pals*" OR "central paralys*" OR "cerebral paralys*" OR "cerebral pares*" OR (encephalopathi* AND infantil*) OR "spastic diplegi*" OR "little disease*" OR "little's disease*") | 283703 |
| #1 | DE "Language Development" OR DE "Language Delay" OR DE "Delayed Speech" OR DE "Communication" OR (TI (Language OR verbal OR communicat*) OR AB (Language OR verbal OR communicat*)) AND (TI (development* OR training OR learning OR function* OR participat* OR abilit*) OR AB (development* OR training OR learning OR function* OR participat* OR abilit*)) | 8209 |

Search strategy in the Cochrane Library December 2, 2019 (read from bottom-up).

| **Set** | **Search terms** | **Result** |
| --- | --- | --- |
| #3 | #1 AND #2 | 266 |
| #2 | "cerebral pals*" OR "brain pals*" OR "brain paralys*" OR "central pals*" OR "central paralys*" OR "cerebral paralys*" OR "cerebral pares*" OR (encephalopathi* AND infantil*) OR "spastic diplegi*" OR "little disease*" OR "little's disease*" | 27067 |
| #1 | ((Language OR verbal OR communicat*) AND (development* OR training OR learning OR function* OR participat* OR abilit*)) | 3219 |
