# Supplementary material for: Factors associated with spoken language comprehension in children with cerebral palsy: a systematic review
Source: Dev Med Child Neurol. 2020 Aug 27;62(12):1363–73. doi: 10.1111/dmcn.14651 (PMC7692918; doi:10.1111/dmcn.14651)
Supplement: Supplementary file 2 — Appendix S2: Quality assessment. [file DMCN-62-1363-s002.docx]

**Appendix S2**

Quality assessment based on Perera ^63^ and Downs and Black^37^.

*Checklist for measuring study quality*

Study design

Based on Perera ^63^.

| **Level** | **Therapy** | **Prognosis** | **Diagnosis** | **Aetiology** | **Classification** |
| --- | --- | --- | --- | --- | --- |
| I | Systematic review | Systematic review | Systematic review | Systematic review | 4 |
| II | RCT | Inception cohort | Cross-sectional (consecutive) | Prospective cohort | 3 |
| III | Non-randomized experimental | Untreated control patients in RCT | Cross-sectional (non-consecutive) | Retrospective cohort | 2 |
|  | Comparative with concurrent control group | Retrospective cohort | Diagnostic case-control | Case-control/Ecological | 2 |
| IV | Case series | Cohort patients different disease | Case series | Cross-sectional | 1 |

Reporting

Based on Downs and Black ^37^.

1. Is the hypothesis/aim/objective of the study clearly described?

| yes | 1 |
| --- | --- |
| no | 0 |

2. Are the main outcomes to measured clearly described in the Introduction or Methods section?

If the main outcomes are first mentioned in the Results section, the question should be answered no.

| yes | 1 |
| --- | --- |
| no | 0 |

3. Are the characteristics of the patients included in the study clearly described?

In cohort studies and trials, inclusion and/or exclusion criteria should be given. In case-control studies, a case-definition and the source for controls should be given.

| yes | 1 |
| --- | --- |
| no | 0 |

6. Are the main findings of the study clearly described?

Simple outcome data (including denominators and numerators) should be reported for all major findings so that the reader can check the major analyses and conclusions. (This question does not cover statistical tests which are considered below.)

| yes | 1 |
| --- | --- |
| no | 0 |

7. Does the study provide estimates of the random variability in the data for the main outcomes?

In non-normally distributed data the inter-quartile range of results should be reported. In normally distributed data the standard error, standard deviation or confidence intervals should be reported. If the distribution of the data is not described, it must be assumed that the estimates used were appropriate and the question should be answered yes.

| yes | 1 |
| --- | --- |
| no | 0 |

10. Have actual probability values been reported (e.g. 0.035 rather than <0.05) for the main outcomes except where the probability value is less than 0.001?

| yes | 1 |
| --- | --- |
| no | 0 |

External validity

All the following criteria attempt to address the representativeness of the findings of the study and whether they may be generalized to the population from which the study subjects were derived.

11. Were the subjects asked to participate in the study representative of the entire population from which they were recruited?

The study must identify the source population for patients and describe how the patients were selected. Patients would be representative if they comprised the entire source population, an unselected sample of consecutive patients, or a random sample. Random sampling is only feasible where a list of all members of the relevant population exists. Where a study does not report the proportion of the source population from which the patients are derived, the question should be answered as unable to determine.

| yes | 1 |
| --- | --- |
| no | 0 |
| Unable to determine | 0 |

12. Were those subjects who were prepared to participate representative of the entire population from which they were recruited?

The proportion of those asked who agreed should be stated. Validation that the sample was representative would include demonstrating that the distribution of the main confounding factors was the same in the study sample and the source population.

| yes | 1 |
| --- | --- |
| no | 0 |
| Unable to determine | 0 |

Internal validity – bias

16. If any of the results of the study were based on “data dredging”, was this made clear?

Any analyses that had not been planned at the outset of the study should be clearly

indicated. If no retrospective unplanned subgroup analyses were reported, then

answer yes.

| yes | 1 |
| --- | --- |
| no | 0 |
| Unable to determine | 0 |

17. In trials and cohort studies, do the analyses adjust for different lengths of follow-up of patients, or in case-control studies, is the time period between the intervention and outcome the same for cases and controls ?

Where follow-up was the same for all study patients the answer should yes. If different lengths of follow-up were adjusted for by, for example, survival analysis the answer should be yes. Studies where differences in follow-up are ignored should be answered no.

| yes | 1 |
| --- | --- |
| no | 0 |
| Unable to determine | 0 |
| Not applicable | - |

18. Were the statistical tests used to assess the main outcomes appropriate?

The statistical techniques used must be appropriate to the data. For example nonparametric

methods should be used for small sample sizes. Where little statistical analysis has been undertaken but where there is no evidence of bias, the question should be answered yes. If the distribution of the data (normal or not) is not described it must be assumed that the estimates used were appropriate and the question should be answered yes.

| yes | 1 |
| --- | --- |
| no | 0 |
| Unable to determine | 0 |

20. Were the main outcome measures used accurate (valid and reliable)?

For studies where the outcome measures are clearly described, the question should

be answered yes. For studies which refer to other work or that demonstrates the

outcome measures are accurate, the question should be answered as yes.

Power

Did the study have sufficient power to detect a clinically important effect where the probability

value for a difference being due to chance is less than 5%?

Sample sizes have been calculated to detect a difference of x% and y%.

| yes | 1 |
| --- | --- |
| no | 0 |
| Not reported | 0 |
