# Supplementary material for: Factors associated with spoken language comprehension in children with cerebral palsy: a systematic review
Source: Dev Med Child Neurol. 2020 Aug 27;62(12):1363–73. doi: 10.1111/dmcn.14651 (PMC7692918; doi:10.1111/dmcn.14651)
Supplement: Supplementary file 3 — Appendix S3: Results of the quality assessment. [file DMCN-62-1363-s003.docx]

**Appendix S3**

| **Article** | **Year** | **Design** | **Design classification** | **Items** | | | | | | | | | | | | | **Funding** | **Conflicts of interest** | **Total scores** | | **Quality level** |
| --- | --- | --- | --- | --- | --- | --- | --- | --- | --- | --- | --- | --- | --- | --- | --- | --- | --- | --- | --- | --- | --- |
|  |  |  | *Based on Perera* ^34^ | Reporting | | | | | | External validity | | Internal validity - bias | | | | Power |  |  |  |  |  |
|  |  |  |  | Clearly described hypothesis/aim/objective. | Main outcomes to be measured are clearly described in the Introduction or Methods section. | Clearly described characteristics of the patients included. | Clearly described main findings. | Estimates of the random variability in the data for the main outcomes are provided. | Actual probability values are reported for the main outcomes (except where the probability value is less than 0.001). | Subjects asked to participate in the study are representative of the entire population from which they were recruited. | Subjects who were prepared to participate are representative of the entire population from which they were recruited. | If any of the results of the study were based on “data dredging”, this was made clear. | In trials and cohort studies, the analyses are adjusted for different lengths of follow-up of patients, or in case-control studies, the time period between the intervention and outcome is the same for cases and controls. | The statistical tests used to assess the main outcomes are appropriate. | The main outcome measures used are accurate (valid and reliable). | Sufficient power to detect a clinically important effect is given. | Funding sources are described (yes/no) | Are there any conflict of interest? (yes/no/n.r.) | Total score  4+(13 – n.a.) = potential total score | Percentage (%) | Excellent: 91-100%  Good:  71-90%  Fair:  51-70%  Poor:  ≤50% |
| Bishop et al. | 1990 | Cross-sectional study | 1 | 0 | 1 | 1 | 1 | 1 | 0 | 0 (u.t.d.) | 0 (u.t.d.) | 1 | n.a. | 0 | 1 | 0 (n.r.) | yes | n.r. | 7/16 | 44 | Poor |
| Byun et al. | 2013 | Retrospective cohort study | 2 | 1 | 1 | 1 | 1 | 1 | 0 | 0 (u.t.d.) | 0 (u.t.d.) | 1 | 1 | 1 | 1 | 0 (n.r.) | yes | no | 11/16 | 69 | Fair |
| Chen et al. | 2009 | Prospective cohort study | 3 | 1 | 1 | 1 | 1 | 1 | 1 | 0 | 0 (u.t.d.) | 1 | 1 | 1 | 1 | 0 (n.r.) | no | n.r. | 13/16 | 81 | Good |
| Choi et al. | 2017 | Cross-sectional study | 1 | 1 | 1 | 1 | 1 | 1 | 1 | 1 | 0 (u.t.d.) | 1 | n.a. | 1 | 1 | 0 (n.r.) | no | n.r. | 11/15 | 73 | Good |
| Coleman et al. | 2016 | Cross-sectional study | 1 | 1 | 1 | 1 | 1 | 1 | 1 | 1 | 1 | 1 | n.a. | 1 | 1 | 0 (n.r.) | yes | no | 12/15 | 80 | Good |
| Coleman et al. | 2013 | Prospective cohort study | 3 | 1 | 1 | 1 | 1 | 1 | 1 | 1 | 1 | 1 | 1 | 1 | 1 | 0 (n.r.) | suppliers described | n.r. | 15/16 | 94 | Excellent |
| Critten et al. | 2018 | Cross-sectional study | 1 | 1 | 1 | 1 | 1 | 0 | 0 | 0 | 0 | 1 | n.a. | 1 | 1 | 0 (n.r.) | yes | n.r. | 8/15 | 53 | Fair |
| Geytenbeek et al. | 2015a | Cross-sectional study | 1 | 1 | 1 | 1 | 1 | 1 | 1 | 1 | 0 (u.t.d.) | 1 | n.a. | 1 | 1 | 0 (n.r.) | yes | no | 11/15 | 73 | Good |
| Geytenbeek et al. | 2015b | Cross-sectional study | 1 | 1 | 1 | 1 | 1 | 1 | 1 | 1 | 0 (u.t.d.) | 1 | n.a. | 1 | 1 | 0 (n.r.) | yes | no | 11/15 | 73 | Good |
| Geytenbeek et al. | 2015c | Cross-sectional study | 1 | 1 | 1 | 1 | 1 | 1 | 1 | 1 | 0 (u.t.d.) | 1 | n.a. | 1 | 1 | 0 (n.r.) | yes | no | 11/15 | 73 | Good |
| Heijden-Maessen et al. | 1990 | Cross-sectional study | 1 | 1 | 0 | 0 | 0 | 0 | 1 | 0 | 0 (u.t.d.) | 0 (u.t.d.) | n.a. | 1 | 1 | 0 (n.r.) | no | n.r. | 5/15 | 33 | Poor |
| Holck et al. | 2009 | Cross-sectional study | 1 | 1 | 1 | 1 | 1 | 1 | 1 | 1 | 0 (u.t.d.) | 1 | n.a. | 1 | 1 | 0 (n.r.) | yes | n.r. | 11/15 | 73 | Good |
| Hustad et al. | 2018 | Prospective cohort study | 3 | 1 | 1 | 1 | 1 | 1 | 1 | 0 (u.t.d.) | 0 (u.t.d.) | 1 | 1 | 1 | 1 | 0 (n.r.) | yes | no | 13/16 | 81 | Good |
| Hustad et al. | 2017 | Prospective cohort study | 3 | 1 | 1 | 1 | 1 | 1 | 1 | 1 | 0 (u.t.d.) | 1 | 1 | 1 | 1 | 0 (n.r.) | yes | no | 14/16 | 88 | Good |
| Lee et al. | 2010 | Cross-sectional study | 1 | 1 | 1 | 1 | 1 | 1 | 1 | 1 | 0 (u.t.d.) | 1 | n.a. | 1 | 1 | 0 (n.r.) | no | n.r. | 11/15 | 73 | Good |
| Lipscombe et al. | 2016 | Prospective cohort study | 3 | 1 | 1 | 1 | 1 | 1 | 1 | 0 | 1 | 1 | 1 | 1 | 1 | 0 (n.r.) | yes | no | 14/16 | 88 | Good |
| Mei et al. | 2016 | Cross-sectional study | 1 | 1 | 1 | 1 | 1 | 1 | 1 | 1 | 1 | 1 | n.a. | 1 | 1 | 0 (n.r.) | yes | no | 12/15 | 80 | Good |
| Nordberg et al. | 2015 | Cross-sectional study | 1 | 1 | 1 | 1 | 1 | 1 | 1 | 1 | 1 | 1 | n.a. | 1 | 1 | 0 (n.r.) | yes | no | 12/15 | 80 | Good |
| Pirila et al. | 2007 | Cross-sectional study | 1 | 1 | 1 | 1 | 1 | 1 | 0 | 0 (u.t.d.) | 0 | 1 | n.a. | 1 | 1 | 0 (n.r.) | yes | n.r. | 9/15 | 60 | Fair |
| Stadskleiv et al. | 2017 | Cross-sectional study | 1 | 1 | 1 | 1 | 1 | 1 | 1 | 1 | 1 | 1 | n.a. | 1 | 1 | 0 (n.r.) | no | no | 12/15 | 80 | Good |
| Vos et al. | 2014 | Prospective cohort study | 3 | 1 | 1 | 1 | 1 | 1 | 1 | 1 | 1 | 1 | 1 | 1 | 1 | 0 (n.r.) | yes | no | 15/16 | 94 | Excellent |

n.a.: not applicable; u.t.d.: unable to determine; n.r.: not reported
