# Supplementary material for: Factors associated with spoken language comprehension in children with cerebral palsy: a systematic review
Source: Dev Med Child Neurol. 2020 Aug 27;62(12):1363–73. doi: 10.1111/dmcn.14651 (PMC7692918; doi:10.1111/dmcn.14651)
Supplement: Supplementary file 4 — Figure S1: Flowchart of search and selection process. [file DMCN-62-1363-s004.docx]

Figure S1 Flowchart of search and selection process

Records identified through database searching
(n = 4224)

## Screening

## Included

## Eligibility

## Identification

Additional records identified through other sources
(n = 24)

Records after duplicates removed
(n = 2898)

Records screened
(n = 2898)

Records excluded
(n = 2826)

Full-text articles assessed for eligibility
(n = 72)

Full-text articles excluded, with reasons
(n = 51)

No specification of spoken language comprehension outcomes (n = 20)

No specification of language outcomes in children or adults with CP and/or children with another medical diagnose or TD children (n = 5)

No description of the relation with any personal or environmental factors (n = 7)

No spoken language comprehension tested at all or with a non-standardized test (n = 12)

Reported about overall communication in or overall information about children with CP (n = 6)

Full text was not retrievable (n = 1)

Studies included in qualitative synthesis
(n = 21

Studies included in quantitative synthesis (meta-analysis)
(n = 21)
