# Supplementary material for: Factors associated with spoken language comprehension in children with cerebral palsy: a systematic review
Source: Dev Med Child Neurol. 2020 Aug 27;62(12):1363–73. doi: 10.1111/dmcn.14651 (PMC7692918; doi:10.1111/dmcn.14651)
Supplement: Supplementary file 5 — Table S1: Study characteristics and reported factors [file DMCN-62-1363-s005.docx]

Table S1 Study characteristics and reported factors

|  |  | **Participants** | | | | | | | **Language assessment** | | **Factors** | | | |
| --- | --- | --- | --- | --- | --- | --- | --- | --- | --- | --- | --- | --- | --- | --- |
| **Author** | **Year** | **Number** | **Age** | **Gender** | **Motor type of CP** | **Mobility** | **Epilepsy** | **Speech** | **Test/Scale/Questionnaire** | **ICF-CY code** | **Domain** | **ICF-CY code** | **Measurement instrument** | **Association** |
| Bishop et al. | 1990 | 48 | 10-18 yrs | Not reported | Not reported | Not reported | Not reported | Non-speaking: n=12; Dysartric speaking: n=12; Normal speaking: n=24 | *Test*: Phoneme discrimination task, BPVS & TROG | b16700 | Intellectual functions  Speech functions  Age | b117  b320 | Raven’s Standard Progressive Matrices  Clinical observation | Phon D – Raven: 0.31*  BPVS – Raven: 0.39**  TROG – Raven: 0.42**  Phon D: speech impaired – controls: p<0.001  BPVS: speech-impaired – controls: p<0.05  TROG: speech-impaired – controls: ns  Phon D – Age:  -0.06  BPVS - Age: 0.17  TROG – Age: 0.16 |
| Byun et al. | 2013 | 8 | 16 - 29 mo | Female: n=5; Male: n=3 | Spastic: n=4; dyskinetic: n=3; ataxic: n=1 | GMFCS: I: n=1; III: n=2; V: n=4. BSID II: GMFCS I - III scored 9-13 months; GMFCS V scored < 1-6 months | Not reported | Non-speaking: n=4 (GMFCS V);  Speaking: n=4, (GMFCS I, III) | *Test:* Korean version of the Ling's stage (K-Ling): subsection Phonologic and phonetic development  *Scale*: Sequenced Language Scale for Infants (SELSI): subsections Receptive & Expressive language | b16700  b16700 | Speech functions | b320 | K-Ling’s Stage, subsection: Phonetic development | Missing |
| Chen et al. | 2009 | 46 | 2 - 5 yrs | Female: n=24; Male: n=22 | Bilateral spastic: n=46 | CCDI, subsection: Gross motor | Not reported | Not reported | *Questionnaire:* Chinese Children Developmental Inventory (CCDI), subsection: Concept comprehension | b3100, b3101 | Specific mental functions of expressive language  Arm hand functioning  Mobility  Social skills | b167  d440,445  d450,455, 460,465  d710,720,750 | CCDI  subsections:  Expressive language  Fine motor  Gross motor  Personal social | r value:  0.89**/0.87**  0.71**/0.68**  0.46**/0.37*  0.84**/0.82** |
| Choi et al. | 2017 | 172 | 3-7 yrs | Female: n=55; Male: n=117 | Unilateral: n=41; bilateral; n=131.  Spastic: n=139; dyskinetic: n=21; ataxic: n= 5; mixed: n=7 | GMFCS  I: n=47;  II: n= 43; III: n= 30; IV: n= 26; V: n=26 | No seizure: n=103; Controlled epilepsy without AED: n=22, Controlled epilepsy with AED: n=30; Epilepsy with AED or epileptic surgery: n=17 | Not reported | *Scale:* Sequenced Language Scale for Infants (SELSI) (n=76; age < 3 years, and if language levels were inadequate) or Preschool Receptive-Expressive Language Scale (PRES) (n=96; age > 3 years and older). | b16700 | Structure of brain  Motor type  Motor distribution  Epilepsy  Intellectual functions  Seeing functions  Hearing functions  Mobility | s110  s110  s110  s110  b117  b210  b230  d450,455,460, 465 | Categories of Bax et al. (2006)  Clinical observation  Clinical observation  Clinical observation  Clinical observation  Clinical observation  Clinical observation  GMFCS | brain MRI: p=0.175  PVWL p<0.001/p=0.006  Deep gray matter: p=0.012  p = 0.080  p=0.003  p<0.001  p<0.001  p=0.009  p<0.001  p=0.824 |
| Coleman et al. | 2016 | 131 | 18-36 mo | Female: n=58; Male: n=73 | Unilateral: n= 39, right: n=19, left: n=20; Bilateral: n = 92  Spastic: n=113; all others: n= 18 | GMFCS  I: n=57;  II: n=14; III: n=19; IV: n=17; V: n=24 | Epilepsy: n=38 | Not reported | *Questionnaire:* Communication and Symbolic Behavioural Scales Developmental Profile (CSBS-DP) Infant-Toddler Checklist, subsections: Receptive language and Symbolic skills (symbolic composite) | b16700 | Structure of brain  Mobility | s110  d450,455,460,465 | KM categories (Krägeloh-Mann, 2004)  Severity of lesion (Fiori et al. 2014)  Laterality index (Desmond et al., 1995)  Language Path  GMFCS | p=0.10  p<0.001  p=0.91  p=0.03  p<0.001 |
| Coleman et al. | 2013 | 124 | 24 (+/- SD 1.0) mo | Female: n=41; Male: n=83 | Unilateral: n=34; bilateral: n=90.  Spastic: n=109; all others: n=15. No. of limbs involved: 1: n=3; 2: n=59; 3: n=14; 4: n=48 | GMFCS  I: n=47;  II: n=14; III: n=22; IV: n=19; V: n=22. | Epilepsy: n=29 | Not reported | *Questionnaire:* Communication and Symbolic Behavior Scales Developmental Profile (CSBS-DP) Infant-Toddler Checklist, subsection: Symbolic composite | b16700 | Motor type  Motor distribution (unilateral - bilateral)  Motor distribution (no. of limbs involved)  Epilepsy  Seeing functions  Hearing functions  Mobility  SES  Birth order | s110  s110  s110  s110  b210  b230  d450,455,460,465  e165  e310 | Clinical observation  Clinical observation  Clinical observation  Clinical observation  Clinical observation  Clinical observation  GMFCS  SEIFA disadvantage tertiles  Clinical observation | MD: -2.5, ns  MD: -1.9, p<0.05  1 limb: MD: -2.3, ns  2 limbs: ref.  3 limbs: MD: -2.7, p<0.05)  4 limbs: MD: -5.2, P<0.05)  MD: -3.5, p<0.05  MD: -2.6, p<0.05  MD: -2.2, ns  I: reference  II: MD: -2.0, p=0.05  III: MD: -3.3, p<0.01  IV: MD: -5.5, p<0.01  V: MD:-6.7, p<0.01  Missing  MD: 3.0, p<0.05 |
| Critten et al. | 2018 | 15 | 6;9 – 11;6 yrs | Female: n=5;  Male: n=10 | Spastic hemiplegia: n=3; Spastic quadriplegia: n=6; Athetoid: n=1; Unknown: n=5 | Wheelchair user: n=2; Not described: n=13 | Not reported | Slow speech: n=7; Occasional stammer: n=1; Selective mutism: n=1; Not described: n=6 | *Test:* subsection Receptive Vocabulary of DST-J (Dyslexia Screening Test, Junior) | b16700 | Reading skills | d166 | Several subsections of DST-J, Salford Reading Test and Thames Valley Test Company (TVTC) | - |
| Geytenbeek et al. | 2015a | 68 | 1;7 - 12;0 yrs | Female: n=33; Male: n=35 | Spastic: n=31; Dyskinetic: n=37 | GMFCS  IV: n = 29; V: n = 39. | Epilepsy: n=20; no epilepsy: n=47; missing: n=1. | Anarthria: n=68 (productive vocabulary fewer than five words). | *Test:* Computer-Based instrument for Low motor Language Testing (C-BiLLT) | b16700 | Motor type  Epilepsy  Arm hand functioning  Mobility  Educational level of parents  Age  Gender | s110  s110  d440,d445  d450,455,460,465  e165 | Clinical observation  Clinical observation  MACS  GMFCS  Clinical observation | β=0.52, p<0.001  -  -  β =-0.30, p<0.01  +  β =0.34, p<0.001  - |
| Geytenbeek et al. | 2015b | 87 | 1;9 - 12 yrs | Female: n=43; Male: n=44 | Spastic: n=47; Dyskinetic: n=40 | GMFCS  IV: n=34; V: n=53. | Epilepsy: n=33; no epilepsy: n=53; missing: n=1. | Anarthria: n=87 (productive vocabulary fewer than five words). | *Test*: Computer-Based instrument for Low motor Language Testing (C-BiLLT) | b16700 | Motor type  Epilepsy  Arm hand functioning  Mobility  Age | s110  s110  d440, 445  d450,455,460,465 | Clinical observation  Clinical observation  MACS  GMFCS | β =0.36, p<0.001  -  -  β =-0.30, p<0.001  β =0.065, p<0.001 |
| Geytenbeek et al. | 2015c | 80 | 1;7-12 yrs | Female: n=39; Male: n=41 | Spastic: n= 43; dyskinetic: n=37 | GMFCS  IV: n=30; V: n=50 | Epilepsy: n=31; no epilepsy: n=47 | Anarthria: n=80 (productive vocabulary fewer than five words). | *Test:* Computer-Based instrument for Low motor Language Testing (C-BiLLT) | b16700 | Structure of brain | s110 | MRI classification using Cioni et al. (1997) | F=9.523, p=0.014* |
| Heijden-Maessen, van der et al. | 1990 | 80 | 2 - 18 yrs | Female: n=31; Male: n=49 | Unilateral: Right: n=46;  Left: n=34 | Amount of adaptations made to the leg: 1. no adaptations: n=16;  2. a splint, a socket, a brace or a plaster treatment: n=22;  3. surgical intervention: n=4;  4. operation and at the same time one or more adaptations: n=2. | Not reported | Not reported | *Test:* Reynell Developmental Language Scales, for some children other tests had been used (not reported which tests). | b16700 | Motor distribution  Intellectual functions  Arm hand functioning  Mobility | s110  b117  d440,445  d450,455,460,465 | Not reported  Clinical observation  Classification based on grip and use of hand  Classification based on amount of adaptations of the leg (see Mobility) | X²=0.387, p=0.53  Spearman’s rank correlation: p=0.0002*  Spearman’s rank correlation: for grip; p=0.08, for use; p=0.11  Spearman’s rank correlation:  p=0.48 |
| Holck et al. | 2009 | 10 | 6;0 - 10;6 yrs | Female: n=3; Male: n=7 | Spastic diplegia (bilateral spastic): n=10 | Not reported. | Not reported | Understandable speech: n=10 | *Test:* TROG & PPVT | TROG: b16700  PPVT: b16700 | Reading skills | d166 | Material from Bishop and Adams (1992) | Partial correlations:  Inferential comprehension – TROG: 0.762*  Literal comprehension – PPVT: 0.749* |
| Hustad et al. | 2018 | 85 | 18 – 60 mo | Female: n=43; Male: n=42 | Spastic: hemiplegia: n=25; diplegia: n=14; triplegia: n=1; quadriplegia: n=25; Unknown: n=1; Dystonic: n=3; Choreo-athetotic: n=1; Ataxic: n=4; Mixed: n=5; Unknown: n=6 | GMFCS  I: n=21  II: n=22  III: n=9  IV: n=15  V: n=18 | Not reported | No speech motor involvement at 48-54 mo: n=19;  Speech motor involvement at 48-54mo: n=36;  Anarthria at 48-54: n=30 | *Questionnaire:* Preschool Language Scale, fourth edition (children younger than 36 mo or if the child was not able to participate in TACL)  *Test:* Test of Auditory Comprehension of Language, third edition (children of 36 mo and older) or  Peabody Picture Vocabulary Test, fourth edition (children who could not tolerate the longer TACL, but could participate in picture pointing tasks) | b16700  b16700 | Speech functions | b320 | Clinical observation | Anarthria profile group (ANAR):  χ² = 739.1, p<0.001  Speech motor impairment profile group (SMI):  χ² = 23.1, p<0.001  No speech motor impairment profile group (NSMI):  χ² = 13.1, p=0.001 |
| Hustad et al. | 2017 | 30 | 24 - 53 mo | Female: n=15; Male: n=15 | Spastic: n=30.  Hemiplegia, left: n=4; Hemiplegia, right: n=8;  Diplegia: n=3; Triplegia: n=0; Quadriplegia: n=15; Unknown: n=0 | GMFCS  I: n=8;  II: n=2;  III: n=4;  IV: n=6;  V; n=10 | Not reported | Established talkers: n=4; Emerging talkers: n= 11; Not yet talking: n= 15 | *Test:* Preschool Language Scale - 4 | b16700 | Speech functions | b320 | Test of Children’s Speech (TOCS+) | Fisher’s exact test p=0.0001 |
| Lee et al. | 2010 | CP: 137  TD: 18, matched for age and gender with CP group | CP: 1.6 - 5.8 yrs  TD: 3.3 - 4.7 yrs | CP: Female: n=62; Male: n=75 TD: Female: n=5; Male: n=13 | Spastic: n=137. Diplegia: n = 59; Quadriplegia: n = 78 | CCDI, subsection: Gross motor | Not reported | Not reported | *Questionnaire:* CCDI, subsection Comprehension-conceptual ability (comprehension of language and abstract concepts) | d3100, d3101 | Motor distribution  Specific mental functions of expressive language  Arm hand functioning  Mobility  Social skills | s110  b1671  d440,445  d450,455,460,465  d710,720,750 | Clinical observation  CCDI, subsection:  Expressive language  Fine motor  Gross motor  Personal-social | +  Pearson’s correlation:  0.868**  0.665**  0.773**  0.922** |
| Lipscombe et al. | 2016 | 71 | motor function and communication assessment at ± 24 mo. and social function outcomes at ± 60 mo. | Female: n=28; Male: n=43 | Unilateral spastic: n = 17;  Bilateral spastic: n = 43;  Dystonia: n = 3; Hypotonia: n = 4; Athetosis: n = 3; Unknown/missing: n = 1 | GMFCS  I: n=24;  II: n = 9;  III: n = 12;  IV: n = 10;  V: n = 16 | Not reported | CSBS-DP, subsection: Speech communciation | *Questionnaire:* CSBS-DP infant-toddler checklist. Parent report version was used. Subsection: Symbolic communication | b16700 | Speech functions  Mobility  Social skills | b320  d450,455,460,465  d710,720,750 | CSBS-DP, subsection: Speech communication  GMFM  PEDI, subsection: Social function | 0.60***  0.63***  0.61*** |
| Mei et al. | 2016 | 84 | 5- 6 yrs | Female: n=37; Male: n=47 | Motor type: Spastic (n = 66); Dyskinesia (n = 1); Hypotonia (n = 3); Ataxic (n = 1); Mixed (n = 13); Unknown (n = 0). Distribution: Monoplegia (n = 1); Hemiplegia (n = 32); Diplegia (n = 25); Tripegia (n = 1); Quadriplegia (n = 25); Unknown (n = 0) | GMFCS  I: n=33; II: n=15; III: n=13; IV: n=16; V: n=7; Unknown: n=0 | Epilepsy: n=18; No epilepsy: n=65; Resolved: n=1; Unknown: n=0 | Non-verbal subgroup: n=20; Verbal subgroup: n=64 | *Test*: Preschool Language Scale, Fourth Edition (PLS4) & PPVT  *Questionnaire:* CSBS-DP CQ was used in the non-verbal subgroup and filled in by the parents. | PSL4: b16700  PPVT:  b16700  b16700 | Intellectual functions  Mobility | b117  d450,455,460,465 | Columbia Mental Maturity Scale/VCPR (IQ<70) if unable to complete CMMS  GMFCS | OR 13.4, 95% CI 2.6-68.3, p=0.002  OR 4.4, 95% CI 0.8-24.2, p=0.09 |
| Nordberg et al. | 2015 | 15 | 9;2 – 12;9 yrs | Female: n=7; Male: n=8 | Unilateral spastic CP (n=7); Bilateral spastic CP (n=2); Dyskinetic (n=2); Ataxi (n=3) | GMFCS  I: n=9;  II: n=1;  III: n=2  IV: n=3;  V: n=0 | Not reported | All children had impaired speech | *Test:* TROG & PPVT-IV | TROG: b16700  PPVT-IV: b16700 | Specific mental functions of expressive language | b1671 | Narrative Assessment Profile (NAP) | r=0.719, p=0.03 |
| Pirila et al. | 2006 | 36 | 1;10 – 9;0 yrs | Female: n=16; Male: n=20 | Diplegia with spastic paresis most pronounced in the lower extremities (n=22); hemiplegia (n=5); quadriplegia with the paresis most pronounced in the upper extremities (n=9) | Gross Motor Limitation Scale Mild (I) (comparable to GMFCS I and II): n=12; Moderate (II) (comparable to GMFCS III): n=7; Severe (III) (comparable to GMFCS IV and V): n=17 | Not reported | The assessment of oral motor patterns and structure, rated as: Normal; Immature; Deviant. Phonology and articulation skills were classified as either normal; immature or deviant. | *Test*: Reynell Developmental Language Scale—revised | b16700 | Severity of CP, based on motor score  Intellectual functions | b117 | Gross and fine motor scores were combined into 3 grades: mild, moderate, severe  WPPSI –R (Wechsler Preschool and Primary Scales of Intelligence – Revised) | χ² = 13.31, p<0.01  χ² = 28.08, p<0.001 |
| Stadskleiv et al. | 2017 | 70 | 5;1 - 17;7 yrs | Female: n=38; Male: n=32 | Spastic hemiplegia (n = 35); Spastic diplegia (n = 18); Spastic quadriplegia (n=9); Dyskinesia (n =8) | GMFCS  I: n = 36; II: n=13; III: n=3; IV: n = 10; V: n=8 | Using antiepileptic medication: n=19 | VSS:  I: n = 42; II: n =12; III: n = 5; IV: n = 11 | *Test:*  - 6y and older: British Picture Vocabulary Scale (BPVS-II) - less than 6y or if BPVS was challenging: Receptive Vocabulary from the WPPSI-III. - if raw scores on both Receptive Vocabulary and BPVS-III were 0: TROG-II | b16700  b16700  b16700 | Motor type  &  Motor distribution  Mobility | s110  s110  d450,455,460,465 | Clinical observation  GMFCS | F=0.906, p=0.443  F=4.514, p=0.015* |
| Vos et al. | 2014 | 418 | 0 – 24 yrs | Female: n=157; Male: n=261 | Unilateral spastic: n=161;  Bilateral spastic: n=202; non-spastic (including dyskinetic, ataxic): n=55 | GMFCS  I: n =206; II: n = 57; III: n = 59; IV: n = 54; V: n = 42 | Epilepsy: n=73;  No epilepsy: n=345 | Not reported | *Scale:*  VABS, subsection: Receptive communication | d3100, d3101 | Motor type  Intellectual functions | s110  b117 | Clinical observation  SON-R for toddlers and Raven’s Coloured Progressive Matrices for children | Unilateral spastic CP: 0 ref  Bilateral spastic CP: -1.65 (1.16), ns  Non-spastic CP: -2.31 (2.49), ns  -4.00 (1.16), p<0.01 |

ns: non-significant; ref: reference value; *: p<0.05; **: p<0.01; ***: p<0.001; +: associated, no value given; -: not associated, no value given
