# Supplementary material for: Factors associated with spoken language comprehension in children with cerebral palsy: a systematic review
Source: Dev Med Child Neurol. 2020 Aug 27;62(12):1363–73. doi: 10.1111/dmcn.14651 (PMC7692918; doi:10.1111/dmcn.14651)
Supplement: Supplementary file 6 [file DMCN-62-1363-s006.pdf]

Table S1 Study characteristics and reported factors

		Participants							Language assessment		Factors			
**Author**	**Year**	**Number**	**Age**	**Gender**	**Motor type of CP**	**Mobility**	**Epilepsy**	**Speech**	**Test/Scale/Questionnaire**	**ICF-CY code**	**Domain**	**ICF-CY code**	**Measurement instrument**	**Association**
Bishop et al.	1990	48	10-18 yrs	Not reported	Not reported	Not reported	Not reported	Non-speaking: n=12; Dysartric speaking: n=12; Normal speaking: n=24	*Test*: Phoneme discrimination task, BPVS & TROG	b16700	Intellectual functions	b117	Raven's Standard Progressive Matrices	Phon D – Raven: 0.31* BPVS – Raven: 0.39** TROG – Raven: 0.42**
											Speech functions	b320	Clinical observation	Phon D: speech impaired – controls: p<0.001<br><br>BPVS: speech-impaired – controls: p<0.05<br><br>TROG: speech-impaired – controls: ns
											Age			Phon D – Age: -0.06 BPVS - Age: 0.17 TROG – Age: 0.16
Byun et al.	2013	8	16 - 29 mo	Female: n=5; Male: n=3	Spastic: n=4; dyskinetic: n=3; ataxic: n=1	GMFCS: I: n=1; III: n=2; V: n=4. BSID II: GMFCS	Not reported	Non-speaking: n=4 (GMFCS V); Speaking: n=4, (GMFCS I, III)	*Test:* Korean version of the Ling's stage (K-Ling): subsection Phonologic and	b16700	Speech functions	b320	K-Ling's Stage, subsection: Phonetic development	Missing

						I - III scored 9-13 months; GMFCS V scored < 1-6 months			phonetic development<br>*Scale*: Sequenced Language Scale for Infants (SELSI): subsections Receptive & Expressive language	b16700				
Chen et al.	2009	46	2 - 5 yrs	Female: n=24; Male: n=22	Bilateral spastic: n=46	CCDI, subsection: Gross motor	Not reported	Not reported	*Questionnaire:* Chinese Children Developmental Inventory (CCDI), subsection: Concept comprehension	b3100, b3101	Specific mental functions of expressive language	b167	CCDI subsections:<br>Expressive language	r value:<br>0.89**/0.87**
											Arm hand functioning	d440,445	Fine motor	0.71**/0.68**
											Mobility	d450,455, 460,465	Gross motor	0.46**/0.37*
											Social skills	d710,720,750	Personal social	0.84**/0.82**
Choi et al.	2017	172	3-7 yrs	Female: n=55; Male: n=117	Unilateral: n=41; bilateral; n=131. Spastic: n=139; dyskinetic: n=21; ataxic: n= 5; mixed: n=7	GMFCS<br>I: n=47;<br>II: n= 43;<br>III: n= 30;<br>IV: n= 26;<br>V: n=26	No seizure: n=103; Controlled epilepsy without AED: n=22, Controlled epilepsy with AED: n=30; Epilepsy with AED or epileptic	Not reported	*Scale:* Sequenced Language Scale for Infants (SELSI) (n=76; age < 3 years, and if language levels were inadequate) or Preschool Receptive-Expressive Language Scale (PRES) (n=96; age > 3 years and older).	b16700	Structure of brain	s110	Categories of Bax et al. (2006)	brain MRI: p=0.175<br>PVWL p<0.001/p=0.006<br>Deep gray matter: p=0.012
											Motor type	s110	Clinical observation	p = 0.080
											Motor distribution	s110	Clinical observation	p=0.003
											Epilepsy	s110		p<0.001

							surgery: n=17				Intellectual functions<br>Seeing functions<br>Hearing functions<br>Mobility	b117<br>b210<br>b230<br>d450,455,460, 465	Clinical observation<br>Clinical observation<br>Clinical observation<br>Clinical observation<br>GMFCS	p<0.001<br>p=0.009<br>p<0.001<br>p=0.824
Coleman et al.	2016	131	18-36 mo	Female: n=58; Male: n=73	Unilateral: n= 39, right: n=19, left: n=20; Bilateral: n = 92 Spastic: n=113; all others: n= 18	GMFCS I: n=57; II: n=14; III: n=19; IV: n=17; V: n=24	Epilepsy: n=38	Not reported	*Questionnaire:* Communication and Symbolic Behavioural Scales Developmental Profile (CSBS-DP) Infant-Toddler Checklist, subsections: Receptive language and Symbolic skills (symbolic composite)	b16700	Structure of brain<br>Mobility	s110<br>d450,455,460,465	KM categories (Krägeloh-Mann, 2004)<br>Severity of lesion (Fiori et al. 2014)<br>Laterality index (Desmond et al., 1995)<br>Language Path<br>GMFCS	p=0.10<br>p<0.001<br>p=0.91<br>p=0.03<br>p<0.001
Coleman et al.	2013	124	24 (+/- SD 1.0) mo	Female: n=41; Male: n=83	Unilateral: n=34; bilateral: n=90. Spastic: n=109; all others: n=15.	GMFCS I: n=47; II: n=14; III: n=22; IV: n=19; V: n=22.	Epilepsy: n=29	Not reported	*Questionnaire:* Communication and Symbolic Behavior Scales Developmental Profile (CSBS-DP) Infant-Toddler	b16700	Motor type<br>Motor distribution	s110<br>s110	Clinical observation<br>Clinical observation	MD: -2.5, ns<br>MD: -1.9, $p<0.05$

					No. of limbs involved: 1: n=3; 2: n=59; 3: n=14; 4: n=48				Checklist, subsection: Symbolic composite		(unilateral - bilateral)			
											Motor distribution (no. of limbs involved)	s110	Clinical observation	1 limb: MD: -2.3, ns<br>2 limbs: ref.<br>3 limbs: MD: -2.7, p<0.05)<br>4 limbs: MD: -5.2, P<0.05)
											Epilepsy	s110	Clinical observation	MD: -3.5, p<0.05
											Seeing functions	b210	Clinical observation	MD: -2.6, p<0.05
											Hearing functions	b230	Clinical observation	MD: -2.2, ns
											Mobility	d450,455,460,465	GMFCS	I: reference<br>II: MD: -2.0, p=0.05<br>III: MD: -3.3, p<0.01<br>IV: MD: -5.5, p<0.01<br>V: MD:-6.7, p<0.01
											SES	e165	SEIFA disadvantage tertiles	Missing
											Birth order	e310	Clinical observation	MD: 3.0, p<0.05
Critten et al.	2018	15	6;9 – 11;6 yrs	Female: n=5; Male: n=10	Spastic hemiplegia: n=3; Spastic quadriplegia: n=6; Athetoid:	Wheelchair user: n=2; Not described: n=13	Not reported	Slow speech: n=7; Occasional stammer: n=1; Selective	*Test:* subsection Receptive Vocabulary of DST-J (Dyslexia Screening Test, Junior)	b16700	Reading skills	d166	Several subsections of DST-J, Salford Reading Test	-

					n=1; Unknown: n=5			mutism: n=1; Not described: n=6					and Thames Valley Test Company (TVTC)	
Geytenbeek et al.	2015a	68	1;7 - 12;0 yrs	Female: n=33; Male: n=35	Spastic: n=31; Dyskinetic: n=37	GMFCS IV: n = 29; V: n = 39.	Epilepsy: n=20; no epilepsy: n=47; missing: n=1.	Anarthria: n=68 (productive vocabulary fewer than five words).	*Test:* Computer-Based instrument for Low motor Language Testing (C-BiLLT)	b16700	Motor type	s110	Clinical observation	β=0.52, p<0.001
											Epilepsy	s110	Clinical observation	-
											Arm hand functioning	d440,d445	MACS	-
											Mobility	d450,455,460,465	GMFCS	β =-0.30, p<0.01
											Educational level of parents	e165	Clinical observation	+
											Age			β =0.34, p<0.001
											Gender			-
Geytenbeek et al.	2015b	87	1;9 - 12 yrs	Female: n=43; Male: n=44	Spastic: n=47; Dyskinetic: n=40	GMFCS IV: n=34; V: n=53.	Epilepsy: n=33; no epilepsy: n=53; missing: n=1.	Anarthria: n=87 (productive vocabulary fewer than five words).	*Test*: Computer-Based instrument for Low motor Language Testing (C-BiLLT)	b16700	Motor type	s110	Clinical observation	β =0.36, p<0.001
											Epilepsy	s110	Clinical observation	-
											Arm hand functioning	d440, 445	MACS	-
											Mobility	d450,455,460,465	GMFCS	β =-0.30, p<0.001

											Age			β =0.065, p<0.001
Geytenbeek et al.	2015c	80	1;7-12 yrs	Female: n=39; Male: n=41	Spastic: n= 43; dyskinetic: n=37	GMFCS IV: n=30; V: n=50	Epilepsy: n=31; no epilepsy: n=47	Anarthria: n=80 (productive vocabulary fewer than five words).	*Test:* Computer-Based instrument for Low motor Language Testing (C-BiLLT)	b16700	Structure of brain	s110	MRI classification using Cioni et al. (1997)	F=9.523, p=0.014*
Heijden-Maessen, van der et al.	1990	80	2 - 18 yrs	Female: n=31; Male: n=49	Unilateral: Right: n=46; Left: n=34	Amount of adaptations made to the leg: 1. no adaptations: n=16; 2. a splint, a socket, a brace or a plaster treatment: n=22; 3. surgical intervention: n=4; 4. operation and at the same time one or more adaptations: n=2.	Not reported	Not reported	*Test:* Reynell Developmental Language Scales, for some children other tests had been used (not reported which tests).	b16700	Motor distribution<br><br>Intellectual functions<br><br>Arm hand functioning<br><br>Mobility	s110<br><br>b117<br><br>d440,445<br><br>d450,455,460,465	Not reported<br><br>Clinical observation<br><br>Classification based on grip and use of hand<br><br>Classification based on amount of adaptations of the leg (see Mobility)	X²=0.387, p=0.53<br><br>Spearman's rank correlation: p=0.0002*<br><br>Spearman's rank correlation: for grip; p=0.08, for use; p=0.11<br><br>Spearman's rank correlation: p=0.48
Holck et al.	2009	10	6;0 - 10;6 yrs	Female: n=3; Male: n=7	Spastic diplegia (bilateral spastic): n=10	Not reported.	Not reported	Understandable speech: n=10	*Test:* TROG & PPVT	TROG: b16700<br>PPVT: b16700	Reading skills	d166	Material from Bishop and Adams (1992)	Partial correlations: Inferential comprehension – TROG: 0.762*<br>Literal comprehension – PPVT: 0.749*

Hustad et al.	2018	85	18 – 60 mo	Female: n=43; Male: n=42	Spastic: hemiplegia: n=25; diplegia: n=14; triplegia: n=1; quadriplegia: n=25; Unknown: n=1; Dystonic: n=3; Choreo-athetotic: n=1; Ataxic: n=4; Mixed: n=5; Unknown: n=6	GMFCS<br>I: n=21<br>II: n=22<br>III: n=9<br>IV: n=15<br>V: n=18	Not reported	No speech motor involvement at 48-54 mo: n=19; Speech motor involvement at 48-54mo: n=36; Anarthria at 48-54: n=30	*Questionnaire:* Preschool Language Scale, fourth edition (children younger than 36 mo or if the child was not able to participate in TACL)<br>*Test:* Test of Auditory Comprehension of Language, third edition (children of 36 mo and older) or Peabody Picture Vocabulary Test, fourth edition (children who could not tolerate the longer TACL, but could participate in picture pointing tasks)	b16700<br><br>b16700	Speech functions	b320	Clinical observation	Anarthria profile group (ANAR): χ^2 = 739.1, p<0.001<br><br>Speech motor impairment profile group (SMI): χ^2 = 23.1, p<0.001<br><br>No speech motor impairment profile group (NSMI): χ^2 = 13.1, p=0.001
Hustad et al.	2017	30	24 - 53 mo	Female: n=15; Male: n=15	Spastic: n=30. Hemiplegia, left: n=4; Hemiplegia, right: n=8; Diplegia: n=3; Triplegia: n=0; Quadriplegia: n=15; Unknown: n=0	GMFCS<br>I: n=8;<br>II: n=2;<br>III: n=4;<br>IV: n=6;<br>V; n=10	Not reported	Established talkers: n=4; Emerging talkers: n= 11; Not yet talking: n= 15	*Test:* Preschool Language Scale - 4	b16700	Speech functions	b320	Test of Children's Speech (TOCS+)	Fisher's exact test p=0.0001
Lee et al.	2010	CP: 137<br>TD: 18, matched for age and gender with CP group	CP: 1.6 - 5.8 yrs<br>TD: 3.3 - 4.7 yrs	CP: Female: n=62; Male: n=75<br>TD: Female: n=5; Male: n=13	Spastic: n=137. Diplegia: n = 59; Quadriplegia: n = 78	CCDI, subsection: Gross motor	Not reported	Not reported	*Questionnaire:* CCDI, subsection Comprehension-conceptual ability (comprehension of language and abstract concepts)	d3100, d3101	Motor distribution<br><br>Specific mental functions of expressive language	s110<br><br>b1671	Clinical observation<br><br>CCDI, subsection: Expressive language	+<br><br>Pearson's correlation: 0.868**

											Arm hand functioning	d440,445	Fine motor	0.665**
											Mobility	d450,455,460,465	Gross motor	0.773**
											Social skills	d710,720,750	Personal-social	0.922**
Lipscombe et al.	2016	71	motor function and communication assessment at ± 24 mo. and social function outcomes at ± 60 mo.	Female: n=28; Male: n=43	Unilateral spastic: n = 17; Bilateral spastic: n = 43; Dystonia: n = 3; Hypotonia: n = 4; Athetosis: n = 3; Unknown/missing: n = 1	GMFCS<br>I: n=24;<br>II: n = 9;<br>III: n = 12;<br>IV: n = 10;<br>V: n = 16	Not reported	CSBS-DP, subsection: Speech communciation	*Questionnaire:* CSBS-DP infant-toddler checklist. Parent report version was used. Subsection: Symbolic communication	b16700	Speech functions	b320	CSBS-DP, subsection: Speech communication	0.60***
											Mobility	d450,455,460,465	GMFM	0.63***
											Social skills	d710,720,750	PEDI, subsection: Social function	0.61***
Mei et al.	2016	84	5- 6 yrs	Female: n=37; Male: n=47	Motor type: Spastic (n = 66); Dyskinesia (n = 1); Hypotonia (n = 3); Ataxic (n = 1); Mixed (n = 13); Unknown (n = 0). Distribution: Monoplegia (n = 1); Hemiplegia (n = 32); Diplegia (n = 25); Tripegia (n = 1); Quadriplegia (n = 25); Unknown (n = 0)	GMFCS<br>I: n=33;<br>II: n=15;<br>III: n=13;<br>IV: n=16;<br>V: n=7;<br>Unknown: n=0	Epilepsy: n=18; No epilepsy: n=65; Resolved: n=1; Unknown: n=0	Non-verbal subgroup: n=20; Verbal subgroup: n=64	*Test*: Preschool Language Scale, Fourth Edition (PLS4) & PPVT *Questionnaire:* CSBS-DP CQ was used in the non-verbal subgroup and filled in by the parents.	PSL4: b16700<br>PPVT: b16700<br>b16700	Intellectual functions	b117	Columbia Mental Maturity Scale/VCPR (IQ<70) if unable to complete CMMS	OR 13.4, 95% CI 2.6-68.3, p=0.002
											Mobility	d450,455,460,465	GMFCS	OR 4.4, 95% CI 0.8-24.2, p=0.09

Nordberg et al.	2015	15	9;2 – 12;9 yrs	Female: n=7; Male: n=8	Unilateral spastic CP (n=7); Bilateral spastic CP (n=2); Dyskinetic (n=2); Ataxi (n=3)	GMFCS<br>I: n=9;<br>II: n=1;<br>III: n=2<br>IV: n=3;<br>V: n=0	Not reported	All children had impaired speech	*Test:* TROG & PPVT-IV	TROG: b16700<br>PPVT-IV: b16700	Specific mental functions of expressive language	b1671	Narrative Assessment Profile (NAP)	r=0.719, p=0.03
Pirila et al.	2006	36	1;10 – 9;0 yrs	Female: n=16; Male: n=20	Diplegia with spastic paresis most pronounced in the lower extremities (n=22); hemiplegia (n=5); quadriplegia with the paresis most pronounced in the upper extremities (n=9)	Gross Motor Limitation Scale<br>Mild (I) (comparable to GMFCS I and II): n=12;<br>Moderate (II) (comparable to GMFCS III): n=7;<br>Severe (III) (comparable to GMFCS IV and V): n=17	Not reported	The assessment of oral motor patterns and structure, rated as:<br>Normal;<br>Immature;<br>Deviant.<br>Phonology and articulation skills were classified as either normal; immature or deviant.	*Test*: Reynell Developmental Language Scale—revised	b16700	Severity of CP, based on motor score<br><br>Intellectual functions	 <br>b117	Gross and fine motor scores were combined into 3 grades: mild, moderate, severe<br><br>WPPSI –R (Wechsler Preschool and Primary Scales of Intelligence – Revised)	χ^2 = 13.31, p<0.01<br><br>χ^2 = 28.08, p<0.001
Stadskleiv et al.	2017	70	5;1 - 17;7 yrs	Female: n=38; Male: n=32	Spastic hemiplegia (n = 35);<br>Spastic diplegia (n = 18);<br>Spastic quadriplegia (n=9);<br>Dyskinesia (n =8)	GMFCS<br>I: n = 36;<br>II: n=13;<br>III: n=3;<br>IV: n = 10;<br>V: n=8	Using antiepileptic medication: n=19	VSS:<br>I: n = 42;<br>II: n =12;<br>III: n = 5;<br>IV: n = 11	*Test:*<br>- 6y and older: British Picture Vocabulary Scale (BPVS-II)<br>- less than 6y or if BPVS was challenging: Receptive Vocabulary from the WPPSI-III.<br>- if raw scores on both Receptive Vocabulary and	b16700<br><br>b16700<br><br>b16700	Motor type &<br>Motor distribution<br><br>Mobility	s110<br>s110<br><br>d450,455,460,465	Clinical observation<br><br>GMFCS	F=0.906, p=0.443<br><br>F=4.514, p=0.015*

									BPVS-III were 0: TROG-II				
Vos et al.	2014	418	0 – 24 yrs	Female: n=157; Male: n=261	Unilateral spastic: n=161; Bilateral spastic: n=202; non-spastic (including dyskinetic, ataxic): n=55	GMFCS I: n =206; II: n = 57; III: n = 59; IV: n = 54; V: n = 42	Epilepsy: n=73; No epilepsy: n=345	Not reported	*Scale:* VABS, subsection: Receptive communication	d3100, d3101	Motor type	s110	Clinical observation	Unilateral spastic CP: 0 ref Bilateral spastic CP: -1.65 (1.16), ns Non-spastic CP: -2.31 (2.49), ns
											Intellectual functions	b117	SON-R for toddlers and Raven's Coloured Progressive Matrices for children	-4.00 (1.16), p<0.01

ns: non-significant; ref: reference value; *: p<0.05; **: p<0.01; ***: p<0.001; +: associated, no value given; -: not associated, no value given
